# Insights into the effects of Friedreich ataxia on the left ventricle using T1 mapping and late gadolinium enhancement

**DOI:** 10.1371/journal.pone.0303969

**Published:** 2024-05-30

**Authors:** Roger E. Peverill, Kimberly Y. Lin, Mark A. Fogel, Michael M. H. Cheung, W. Stuart Moir, Louise A. Corben, Glenn Cahoon, Martin B. Delatycki

**Affiliations:** 1 Monash Cardiovascular Research Centre, MonashHeart and Department of Medicine (School of Clinical Sciences at Monash Health), Monash University and Monash Health, Clayton, Victoria, Australia; 2 Division of Cardiology, Children’s Hospital of Philadelphia, Philadelphia, Pennsylvania, United States of America; 3 Department of Cardiology, Royal Children’s Hospital, Parkville, Victoria, Australia; 4 Department of Paediatrics, University of Melbourne, Parkville, Victoria, Australia; 5 Heart Research Group, Murdoch Children’s Research Institute, Parkville, Victoria, Australia; 6 Bruce Lefroy Centre for Genetic Health Research, Murdoch Children’s Research Institute, Parkville, Victoria, Australia; 7 Victorian Clinical Genetics Services, Parkville, Victoria, Australia; All India Institute of Medical Sciences, INDIA

## Abstract

**Background:**

The left ventricular (LV) changes which occur in Friedreich ataxia (FRDA) are incompletely understood.

**Methods:**

Cardiac magnetic resonance (CMR) imaging was performed using a 1.5T scanner in subjects with FRDA who are homozygous for an expansion of an intron 1 GAA repeat in the *FXN* gene. Standard measurements were performed of LV mass (LVM), LV end-diastolic volume (LVEDV) and LV ejection fraction (LVEF). Native T1 relaxation time and the extracellular volume fraction (ECV) were utilised as markers of left ventricular (LV) diffuse myocardial fibrosis and late gadolinium enhancement (LGE) was utilised as a marker of LV replacement fibrosis. FRDA genetic severity was assessed using the shorter *FXN* GAA repeat length (GAA1).

**Results:**

There were 93 subjects with FRDA (63 adults, 30 children, 54% males), 9 of whom had a reduced LVEF (<55%). A LVEDV below the normal range was present in 39%, a LVM above the normal range in 22%, and an increased LVM/LVEDV ratio in 89% subjects. In adults with a normal LVEF, there was an independent positive correlation of LVM with GAA1, and a negative correlation with age, but no similar relationships were seen in children. GAA1 was positively correlated with native T1 time in both adults and children, and with ECV in adults, all these associations independent of LVM and LVEDV. LGE was present in 21% of subjects, including both adults and children, and subjects with and without a reduced LVEF. None of GAA1, LVM or LVEDV were predictors of LGE.

**Conclusion:**

An association between diffuse interstitial LV myocardial fibrosis and genetic severity in FRDA was present independently of FRDA-related LV structural changes. Localised replacement fibrosis was found in a minority of subjects with FRDA and was not associated with LV structural change or FRDA genetic severity in subjects with a normal LVEF.

## Introduction

Friedreich ataxia (FRDA) is an autosomal recessive neurodegenerative disease caused by pathogenic variants in the *FXN* gene which encodes for the mitochondrial protein frataxin [1]. Cardiac disease is a frequent accompaniment of FRDA, can lead to arrhythmias and cardiac failure, and is the most common cause of death [2–4]. A proportion of subjects with FRDA develop a reduced LV ejection fraction (LVEF) [5–7], and post mortem histology of the heart in FRDA has shown extensive interstitial fibrosis, focal degeneration of muscle fibres and active muscle necrosis [8]. An increase in LV wall thickness and a reduction in LV chamber size are more common than, and likely precede and predispose to, a subsequent reduction of LVEF in FRDA [5–7, 9–15]. However, there is little information about the histological changes in the LV myocardium during the early stages of the disease process.

A better understanding of the changes which occur in the myocardium in FRDA prior to a reduction in LVEF is important for future attempts to modify or prevent cardiac disease progression. Cardiac magnetic resonance (CMR) not only provides a gold standard assessment for LV volumes and LV mass (LVM), but with the use of intravenous gadolinium and late imaging to detect late gadolinium enhancement (LGE), it also provides a non-invasive technique for the visualisation of localised replacement myocardial fibrosis [16]. Furthermore, sensitive quantitative information about more generalised changes in the LV myocardium can be obtained using T1 mapping techniques [17]. Both native T1 relaxation time and calculated myocardial extracellular volume fraction (ECV) have been shown to increase in the presence of diffuse myocardial fibrosis [17–21]. However, there has only been limited investigation in FRDA into the presence of LGE [6, 22–24] or diffuse myocardial fibrosis [24].

Approximately 96% of FRDA is due to homozygosity for a GAA expansion in intron 1 of *FXN*, with the other 4% being compound heterozygous for a GAA expansion and a different *FXN* pathogenic variant. In the homozygous group, the number of GAA repeats in the shorter allele of the *FXN* gene (GAA1) is inversely related to cellular levels of frataxin [25, 26], and there has therefore been interest in the ability of GAA1 to explain cardiac disease severity in FRDA. GAA1 has been reported to be a predictor of cardiac death [3, 4], of progression to a reduced LVEF [7], and has also been associated with increased LV wall thickness [4, 9, 10, 12, 15, 27], relative wall thickness (RWT) [22], LV mass (LVM) [10, 15, 28] and LVM index (LVMI) [9, 29] and a smaller LV end-diastolic diameter (LVEDD) [4, 15] and LV end-diastolic volume (LVEDV) [14, 15]. The aims of the present study using CMR with gadolinium injection in adults and children with FRDA homozygous for GAA repeat expansions, were to quantify LV volumes, LVM, native T1 time and ECV, to determine the presence and extent of LGE, and to investigate the relationship of these LV variables with GAA1, age at onset of symptoms (AOS), symptom duration (SDur), age and the age group (i.e. children versus adults).

## Materials and methods

### Subjects

Children and adults homozygous for a GAA expansion in intron 1 of *FXN* and aged between 10 and 50 years were recruited through clinical research programs (including the Collaborative Clinical Research Network in FA (CCRN-FA) and from the Friedreich Ataxia clinics at Monash Health, Melbourne, Victoria, Australia and the Children’s Hospital of Philadelphia, PA, USA. Prior to undergoing CMR all individuals had their renal function evaluated and estimated glomerular filtration rate (eGFR) calculated to confirm the absence of renal dysfunction and thus the safety of gadolinium administration. Individuals with known ischemic heart disease, hypertension, more than mild valvular disease, a persistent atrial arrhythmia or contraindications to CMR or gadolinium injection were excluded from the study. Approval for this study was provided by the Human Research Ethics Committee of the Royal Children’s Hospital, Melbourne, Victoria, Australia and the Institutional Review Board of the Children’s Hospital of Philadelphia, PA, USA. All participants in the study, or their parents/guardians if the participant was aged under 18 years, provided written informed consent as per the Declaration of Helsinki.

Height and weight were measured, and body surface area (BSA) and body mass index were calculated, using standard formulae. AOS was defined as the time when an individual or their parents first noted neurological or non-neurological symptoms of FRDA, and SDur was defined as the time between AOS and the time of CMR testing. GAA repeat size in the smaller (GAA1) and larger (GAA2) alleles of the *FXN* gene were determined using a single in-house method at the Melbourne site and from different commercial laboratories at the Philadelphia site. Clinical severity of neurological disease was evaluated to provide a neurological profile of the cohort by performance of the neurological component of the Friedreich Ataxia Rating Scale (nFARS) within one month of the CMR study (available in 81 subjects, score out of 125). A blood sample was drawn on the same day as the CMR study for measurement of haematocrit (Hct).

### Cardiac magnetic resonance protocol

CMR studies were performed using similar protocols on 1.5T magnetic resonance imaging units (Magnetom Avanto in Philadelphia and Magnetom Aera in Melbourne; Siemens Medical Solutions, Erlangen, Germany). Breath-held images were acquired in expiration, and a 4-lead vector-electrocardiogram was recorded for gating purposes. Following standard localising images, true fast imaging with steady-state precession (TrueFISP) cine images were acquired in the 4-chamber, 2-chamber, and 3-chamber views and used to plan short axis imaging. End-expiration basal, mid-cavity, and apical short axis native T1 images were collected using the optimised parameter set of a Modified Look-Locker Inversion recovery (MOLLI) research sequence provided by Siemens with an acquisition rest/schema of 5(3)3 designed for long T1 values in native scans.

An IV injection of gadobutrol (Gadovist 1.0, Bayer AG, Berlin, Germany) at a dose of 0.2 mmol/kg body weight was administered to all subjects and immediately following contrast injection, TrueFISP cine functional imaging was obtained in contiguous 7.0 mm slices in the short axis. At 10 minutes post contrast administration a TI scout sequence was used to determine optimal inversion time (average TI = 300 ms) for the late gadolinium enhanced (LGE) images, 8 mm single slice mid ventricle, TR/TE: 23.67/1.1 ms, flip angle 30°, 340 mm FOV, and generated using 20 ms increments from 80 to 500 ms. LGE images were collected using a free breathing phase sensitive inversion recovery (PSIR) technique, as well as single slice, breath held, PSIR with whole ventricular coverage in the short axis. Factors for the two sequences were adjusted to allow direct comparison, typically TR 700 ms, FOV 340 x 280 mm, flip angle 45°, matrix = 256 x 192, TI = 300 ms, voxel size 1.3 x 1.3 x 8.0 mm.

Post contrast T1 mapping sequences were acquired at approximately 15 minutes post contrast injection using an optimised parameter set for MOLLI using the same slice thickness and positions as the native T1 images. TR/TE = 360/112 ms, flip angle = 35°, TI = 260 ms, FOV = 360 x 306 mm, matrix = 256 x 168, interpolated voxel size = 1.4 x 1.4 x 8.0 mm, GRAPPA = 2 with 36 reference lines. An acquisition/rest schema of 4(1)3(1)2 was employed to account for short TI values post contrast.

Standard measurements of CMR volumes, LVM and LVEF, and analysis of T1 maps, were performed at each site using cvi42 v5.3.2 (Circle Cardiovascular Imaging Inc, Calgary, AB, Canada). Papillary muscles were included in the myocardial mass and excluded from LV volumes. The presence of abnormalities of LV end-diastolic volume (LVEDV) and LVM were determined using sex and BSA based criteria specific for children or adults as appropriate, with papillary muscles excluded from LVEDV and included in LVM, and 2 standard deviations (SD) from the mean representing the upper and lower limits of the normal range [30]. Left ventricular hypertrophy (LVH) was defined as a LVM > 2 SD above the mean. A LVEF of <55% was defined as reduced. The LVM volume ratio (LVMVR) was calculated as the ratio of LVM/LVEDV and used as a marker of concentric LV geometry, with an upper limit of the normal range of 0.9 in males and 0.8 in females [30]. For myocardial T1 analysis, manual epicardial and endocardial contours were drawn on the MOLLI mid ventricular short axis slice and segmented according to the American Heart Association (AHA) 16 segment model.

Reported T1 based measurements were an average of results from all the basal and mid LV segments as has been previously described [31]. Extracellular volume fraction (ECV) was calculated using the mean segmental pixel value from the MOLLI ECV maps using the formula: ECV = (Δ[1/T1_myo_]/Δ[1/T1_blood_])*[1-Hct]). A "synthetic ECV" was also calculated using a calculated haematocrit utilising the equation: synthetic haematocrit = 831.6 * (1 / T1_blood_)—0.151 [32]. Native T1 times and ECV in individual FRDA subjects could not be classed into normal or abnormal in this study because of the lack of an adequate comparison group. This is because native T1 times and ECV are not only recognised to be machine and protocol dependent, but also because they are sex- and probably also age-dependent in adults [33–36]. Normal T1 values therefore require large numbers of subjects of different ages and of both sexes, but these were not available for either adults or children for this study. To determine inter-rater reliability of T1 times in CMR studies, 19 randomly chosen scans had T1 measurements repeated at the alternative site, with the study images sent to the alternative site, and the second measurer blinded to the previous measurements.

### Statistical analysis

Results are expressed as mean ± standard deviation or median [range] if not normally distributed. Statistical analysis has been performed using Systat V13 (Systat Software, Chicago, IL, USA). Some of the analyses have been confined to subjects with a normal LVEF because the number of subjects with a reduced LVEF was small, and also because the main focus of this study was on the early myocardial changes which occur in FRDA. Children and adults were compared but also analysed separately because of previous evidence that there may be differences in the relationship of GAA1 with LV structural features based on age group [15]. Univariate linear regression analysis was performed to assess the relationships of CMR variables with selected demographic and FRDA-specific variables of interest based on previous findings about the cardiac effects of FRDA. In particular, BSA, sex, age, age group (child v adult), AOS, SDur and GAA1 were considered in analyses of LV volumes and LVM, and sex [33–38], age [33–36], age group, heart rate [34, 38], AOS, SDur and GAA1 were considered in analyses of T1 mapping variables. The relationship of GAA2 with the above independent variables was also investigated but it was not a predictor of any of the variables and has not been reported in any of the analyses. Categoric variables were included in multivariate models as dummy variables for sex (male = 1, female = 0), age group (adults = 1, children = 0) and study site (Philadelphia = 1, Melbourne = 0). SDur was not normally distributed and was log transformed prior to inclusion in linear regression models. Log SDur was positively correlated with age (r = 0.48, p<0.001) and GAA1 was negatively correlated with AOS (r = -0.58, p<0.001), and therefore these relationships were considered when constructing multivariate models. Independent variables were removed during the multivariate modelling process if the p value was >0.10, but the final models only show independent variables with a p<0.05. Scatter plots and residuals were reviewed to determine if there were outliers, and if identified, the possibility of exclusion of the outlier from the analysis was considered.

## Results

There were 96 subjects with FRDA who underwent CMR imaging, but of these, satisfactory LV volume and mass measurements were not possible in 3 subjects, 2 because of an irregular heart rate during image acquisition, and one because they were found to have been dehydrated at the time of the study. The demographics of the remaining 93 subjects, comprising 63 adults and 30 children, are shown in Table 1. There were three subjects with diabetes, all of whom were adults. The numbers for Melbourne and Philadelphia are shown separately and demonstrate some variations in subject demography between the two sites.

**Table 1:** Demography of study subjects at each study site.

	Total (n = 93)	Philadelphia (n = 56)	Melbourne (n = 37)
Male	50 (53.8%)	28 (50.0%)	22 (59.5%)
Adults	63 (67.7%)	33 (58.9%)	30 (80.6%)
Age (years)	24.1±9.8	20.8±7.4	29.2±10.9
AOS (years)	11.6±5.9	9.8±3.9	14.3±7.4
SDur (years)	8.0 [0–33.8]	5.0 [0.8–26.0]	13.4 [0–33.8]
nFARS score (n = 81)	59±20	63±19	54±20
GAA1	689±223	732±228	623±213
GAA2	921±181	937±188	897±168

AOS—age at onset of symptoms; SDur—symptom duration; nFARS—neurological Friedreich ataxia rating scale score; GAA1 –number of GAA repeats in the smaller allele of the *FXN* gene; GAA2—number of GAA repeats in the larger allele of the *FXN* gene

### Left ventricular mass and volumes

Of the 93 subjects there were 20 (22%) with LVH, this comprising 10/30 (33%) of the children and 10/63 (16%) of the adults. There were 36 subjects (39%) with a small LVEDV, and this comprised 30% of the children and 42% of the adults. There were only 3 subjects who met the criteria for a LVEDV above the normal range, all of whom were adults. There were 9 subjects with a LVEF <55% (6 adults, 3 children), of whom 2 also had LV dilatation and 3 also had an increased LVM. The results of indexed LV volumes, LVMI, LVMVR and LVEF in subjects with a LVEF ≥55% (n = 84) are shown in Table 2, with male and female subjects shown separately. In a comparison of adult subjects with a normal LVEF, LVMI, LVEDVI and LVESVI were all higher in males than females, whereas LVEF was higher in females. LVMVR was greater than the upper limit of the sex-specific normal range in 75/84 (89%) subjects, was higher in children than adults (1.47±0.33 v 1.24±0.38 g/mL, p<0.01), but was similar in adult males and females (p = 0.79).

**Table 2:** Left ventricular indexed volumes and mass in males and females with a normal LVEF.

	Males	Females
N	45 (32 adults)	39 (31 adults)
Age (years)	25.6±10.7	22.0±8.0
Body surface area (m^2^)	1.79±0.33**	1.58±0.24
Body mass index (kg/m^2^)	22.8±4.7	22.0±6.3
LVEDVI (mL/m^2^)	64±13*	57±12
LVESVI (mL/m^2^)	18±8*	14±7
Stroke volume index (mL/m^2^)	46±8	43±8
LVMI (g/m^2^)	83±21**	70±15
LVMVR (g/mL)	1.36±0.40	1.28±0.35
LVEF (%)	72±8*	76±8
Heart rate (/min)	81±14	86±14
Cardiac index (L/min/m^2^)	3.65±0.65	3.69±0.95

LVEDVI—left ventricular end-diastolic volume index; LVESVI—left ventricular end-systolic volume index; LVMI—left ventricular mass index; LVMVR—left ventricular mass volume ratio; LVEF—left ventricular ejection fraction

*p<0.02 and

**p<0.01 for comparison of adult males and females

### Predictors of LVEDV in subjects with a normal LVEF

In subjects with a LVEF ≥55% (n = 84), LVEDV was positively correlated with BSA (r = 0.76, p<0.001) and was higher in males (p<0.001), but male sex was only a borderline significant predictor of LVEDV after adjusting for BSA (p = 0.053). After adjustment for BSA and sex, LVEDV was not different between children and adults (p = 0.70). In adults (n = 57) after adjusting for BSA and sex, AOS was a borderline positive correlate of LVEDV (p = 0.056), but there were no contributions to the model of LVDEV from GAA1 (p = 0.39), log SDur (p = 0.66) or age (p = 0.80). In a multivariate model of LVEDV, BSA and AOS, but not sex, were independent predictors of LVEDV, with an earlier AOS associated with a smaller LVEDV (Table 3). In children (n = 27) BSA was a positive correlate of LVEDV (r = 0.73, p<0.001), and after adjusting for BSA there was no independent contribution from sex to the model (p = 0.80). After including BSA in multivariate models of LVEDV in children there were no contributions from AOS (p = 0.17), GAA1 (p = 0.12) or age (p = 0.15), but there was a borderline significant contribution from log SDur (p = 0.05), with longer disease duration associated with a smaller LVEDV.

**Table 3:** Multivariate model of LVEDV in adult subjects with a normal LVEF.

Independent variable	r value in univariate analysis	*β* in multivariate model	p value in multivariate model	Cumulative adjusted r^2^
BSA	0.67	0.58	<0.001	0.44
Male sex	0.47		NS	0.47
AOS	0.46	0.25	0.019	0.49

BSA—body surface area; AOS—age at the onset of symptoms

### Predictors of LVM in subjects with a normal LVEF

In subjects with a LVEF ≥55% (n = 84), LVM was positively correlated with BSA (p<0.001), larger in males (p<0.001), and both BSA and sex were independent predictors of LVM (p<0.001 for both). After adjustment for BSA and sex, LVM was not different between children and adults (p = 0.11). In adults (n = 57) after including BSA and sex in the model, there were additional contributions from GAA1 (β = 0.36), AOS (β = -0.35) and age (β = -0.39), but not from log SDur (p = 0.57). The model which included GAA1 (Table 4) explained a similar amount of the variance of LVM to the model which contained AOS (Table 5). Neither GAA1 nor AOS remained significant predictors in the model of LVM when both independent variables were included together (p = 0.056 & p = 0.062, respectively). Older age was an independent predictor of a lower LVM in a model which included BSA, sex and GAA1 (Table 4), whereas neither AOS nor age remained significant when age was added to the combination of BSA, sex and AOS (p = 0.067 & p = 0.054, respectively). GAA1 accounted for 10% and age accounted for an additional 5% of the variance in LVM in adults. All the predictors of LVM in Table 4 remained significant after excluding diabetic subjects from the analysis. In children (n = 27) LVM was positively correlated with BSA (r = 0.54), and male sex was a borderline significant predictor of a larger LVM (p = 0.066) after adjusting for BSA. There were no significant contributions to the model of LVM in children which included BSA and sex from any of GAA1 (p = 0.86), AOS (p = 0.97), log SDur (p = 0.79) or age (p = 0.20).

**Table 4:** Multivariate model of LVM including GAA1 in adult subjects with a normal LVEF.

Independent variable	r value in univariate analysis	β in multivariate model	p value in multivariate model	Cumulative adjusted r^2^
BSA	0.62	0.55	<0.001	0.37
Male sex	0.48	0.42	<0.001	0.42
GAA1	0.11	0.28	0.008	0.52
Age	-0.10	-0.27	0.007	0.57

BSA—body surface area, GAA1—number of GAA repeats in the smaller allele of the *FXN* gene

**Table 5:** Multivariate model of LVM including AOS in adult subjects with a normal LVEF.

Independent variable	r value in univariate analysis	β in multivariate model	p value in multivariate model	Cumulative adjusted r^2^
BSA	0.62	0.59	<0.001	0.37
Male sex	0.48	0.36	<0.001	0.42
AOS	-0.01	-0.35	0.001	0.51

BSA—body surface area; AOS—age at the onset of symptoms

### T1 relaxation times

T1 relaxation times pre and post gadolinium contrast, the partition coefficient, and the ECV calculated using both the measured Hct and the calculated Hct, in the total group, and subjects with normal and reduced LVEF, are shown in Table 6. Post contrast T1 imaging was not performed, and thus ECV was also not available, in one of the subjects with a reduced LVEF. In the 87 subjects in whom both methods for ECV calculation could be performed, the synthetic ECV using the calculated Hct gave a slightly higher result (0.286±0.050 v 0.277±0.051, p<0.001), but there was a close correlation between the two methods (r = 0.97, p<0.001). Because it was available in all subjects the synthetic ECV results have been used in all the modelling of ECV. Univariate p values for comparison of normal LVEF with the reduced LVEF group are not provided in Table 6 because there were a number of potential confounding factors including sex and study site (see below).

**Table 6:** T1 relaxation times.

	Total group	LVEF≥55%	LVEF<55%
Native T1 relaxation time (ms) (n = 93)	1012±42	1010±44 (n = 84)	1019±46 (n = 9)
Native blood T1 time (ms) (n = 93)	1569±91	1564±91 (n = 84)	1606±93 (n = 9)
Post contrast T1 time (ms) (n = 92)	476±64	445±64 (n = 84)	415±60 (n = 8)
Post contrast blood T1 time (ms) (n = 92)	314±77	316±80 (n = 84)	300±56 (n = 8)
Partition coefficient (n = 92)	0.47±0.09	0.47±0.07 (n = 84)	0.53±0.13 (n = 8)
Synthetic ECV (n = 92)	0.288±0.051	0.284±0.045 (n = 84)	0.328±0.085 (n = 8)
ECV (n = 87)	0.277±0.051	0.274±0.046 (n = 80)	0.314±0.084 (n = 7)

ECV—extracellular volume fraction; Hct—haematocrit

In univariate analyses of the whole group, native T1 time was not different between the study sites (p = 0.70), but it was higher in women than men (p<0.001) and positively correlated with heart rate (r = 0.30, p = 0.003). After adjusting for sex and heart rate, there was no difference in native T1 time between subjects with a reduced versus a normal LVEF (p = 0.17). Post contrast T1 time and ECV were both higher at the Philadelphia than the Melbourne site (p<0.002 for both). After adjusting for study site, post contrast T1 time was higher in men (p = 0.047), but not correlated with heart rate (p = 0.30) and not different between those with a normal versus reduced LVEF (p = 0.47). After adjusting for study site, ECV was not correlated with heart rate (p = 0.15), there was a trend for it to be higher in females (p = 0.094), and it was higher in subjects with a reduced LVEF (p = 0.001).

### Predictors of native T1 relaxation time in subjects with a normal LVEF

In subjects with a normal LVEF (n = 84), after adjusting for sex, heart rate was a positive correlate of native T1 time (β = 0.21, p = 0.029) and children had a higher native T1 time than adults (p = 0.02), but when included together in a model of native T1 time, neither heart rate (p = 0.09) nor age group (p = 0.061) remained significant predictors. In adults with a normal LVEF (n = 57), after adjusting for sex, GAA1 was an independent positive correlate of native T1 time (β = 0.21, p = 0.013), whereas there were no contributions from any of heart rate (p = 0.30), AOS (p = 0.57), log SDur (p = 0.35), age (p = 0.37), LVMI (p = 0.063) or LVEDVI (p = 0.34). The multivariate model of native T1 time including sex and GAA1 is shown in Table 7. GAA1 accounted for 7% of the variance in native T1 time. There was one outlier with a native T1 time of 861 ms (>3 SD below the group mean), but there was no significant change in the findings when this outlier was excluded from the regression analyses. GAA1 remained a predictor of native T1 time after exclusion of diabetic subjects from the analysis (p = 0.025).

**Table 7:** Multivariate model of native T1 time in adult subjects with a normal LVEF.

Independent variable	r value in univariate analysis	*β* in multivariate model	p value in multivariate model	Cumulative adjusted r^2^
Male sex = 1	-0.55	-0.42	0.001	0.29
GAA1	0.48	0.30	0.013	0.36

GAA1—number of GAA repeats in the smaller allele of the *FXN* gene

In children with a normal LVEF, native T1 time was not higher in females (p = 0.10), GAA1 was a positive correlate of native T1 time (r = 0.45, p = 0.02), but there were no correlations of native T1 time with any of heart rate (p = 0.77), AOS (p = 0.75), log SDur (p = 0.79), age (p = 0.10), LVMI (p = 0.89) or LVEDVI (p = 0.88). GAA1 accounted for 17% of the variance in native T1 time in children.

### Predictors of post contrast T1 relaxation time in subjects with a normal LVEF

In subjects with a normal LVEF (n = 84), after adjusting for study site, male sex was a borderline predictor (p = 0.085) of a higher post contrast T1 time, but there was no difference in post contrast T1 time between children and adults (p = 0.19). When only adult subjects were analysed, however, both study site and sex were independent predictors of post contrast T1 time (Table 8). After adjusting for study site and sex, in adults there were no independent contributions to the prediction of post contrast T1 time from GAA1 (p = 0.16), AOS (p = 0.59), log SDur (p = 0.70), heart rate (p = 0.18), age (p = 0.21), LVMI (p = 0.067) or LVEDVI (p = 0.056). There were no significant predictors of post contrast T1 time in children (p>0.10 for all).

**Table 8:** Multivariate model of post contrast T1 time in adult subjects with a normal LVEF.

Independent variable	r value in univariate analysis	*β* in multivariate model	p value in multivariate model	Cumulative adjusted r^2^
Study site	0.49	-0.50	<0.001	0.22
Male sex = 1	0.33	0.34	0.003	0.33

### Predictors of extracellular volume fraction in subjects with a normal LVEF

In subjects with a normal LVEF (n = 84), ECV was higher at the Philadelphia site (p = 0.002), and after adjustment for study site, it was higher in children than adults (p = 0.013), but not different between males and females (p = 0.11). In contrast, in separate analysis of adults with a normal LVEF (n = 57) after adjustment for study site, female sex was associated with a higher ECV (p = 0.006). In adults after adjustment for study site and sex, ECV was positively correlated with GAA1 (β = 0.30, p = 0.022), there was a positive correlation with log SDur (β = 0.365, p = 0.009), but only a borderline significant contribution from AOS (p = 0.069), and no contributions from heart rate (p = 0.25), age (p = 0.21), LVMI (p = 0.53) or LVEDVI (p = 0.89). A multivariate model of ECV in which there were independent contributions from study site, sex, GAA1 and log SDur is shown in Table 9. GAA1 accounted for 7% of the variance of ECV and log SDur accounted for an additional 7% of the variance in ECV. When diabetics were excluded from the analysis, log SDur remained a significant predictor of ECV (p = 0.038), but GAA1 was no longer significant (p = 0.066). In children there were no significant predictors of ECV from study site, GAA1, AOS, log SDur, age, LVMI and LVEDVI (p>0.10 for all).

**Table 9:** Multivariate model of ECV in adult subjects with a normal LVEF.

Independent variables	r value in univariate analysis	*β* in multivariate model	p value in multivariate model	Cumulative adjusted r^2^
Study site	0.47	0. 47	0.001	0.10
Male sex = 1	-0.35	-0.24	0.050	0.20
GAA1	0.44	0.27	0.032	0.27
Log SDur	0.07	0.34	0.013	0.34

GAA1—number of GAA repeats in the smaller allele of the *FXN* gene; SDur—symptom duration

### Reproducibility of T1 measurements

There were 19 randomly selected CMR studies (8 from Melbourne and 11 from Philadelphia, with gadolinium administered in 18), for which T1 measurements were performed independently by the investigators at both sites. The results of the two measurements are shown in Table 10. There were close correlations of T1 relaxation times for native myocardium and blood (r = 0.96–0.97, n = 19) and for post contrast myocardium and blood (r = 0.96–0.97, n = 18). There were no significant differences in the repeated measurements for any of these variables.

**Table 10:** Blinded remeasurements of T1 relaxation times.

	Native T1 time (ms)	Native blood time (ms)	Post contrast T1 time (ms)	Post contrast blood time (ms)
N	19	19	18	18
Melbourne	1017.3±39.4	1583.3±119.8	439.7±81.3	291.3±94.0
Philadelphia	1014.5±47.4	1574.0±124.3	440.4±74.3	294.8±89.6
p value for comparisons of measurements	0.88	0.50	0.90	0.59
r for correlations between measurements	0.95	0.97	0.96	0.96

### Late gadolinium enhancement

**Fig 1:**
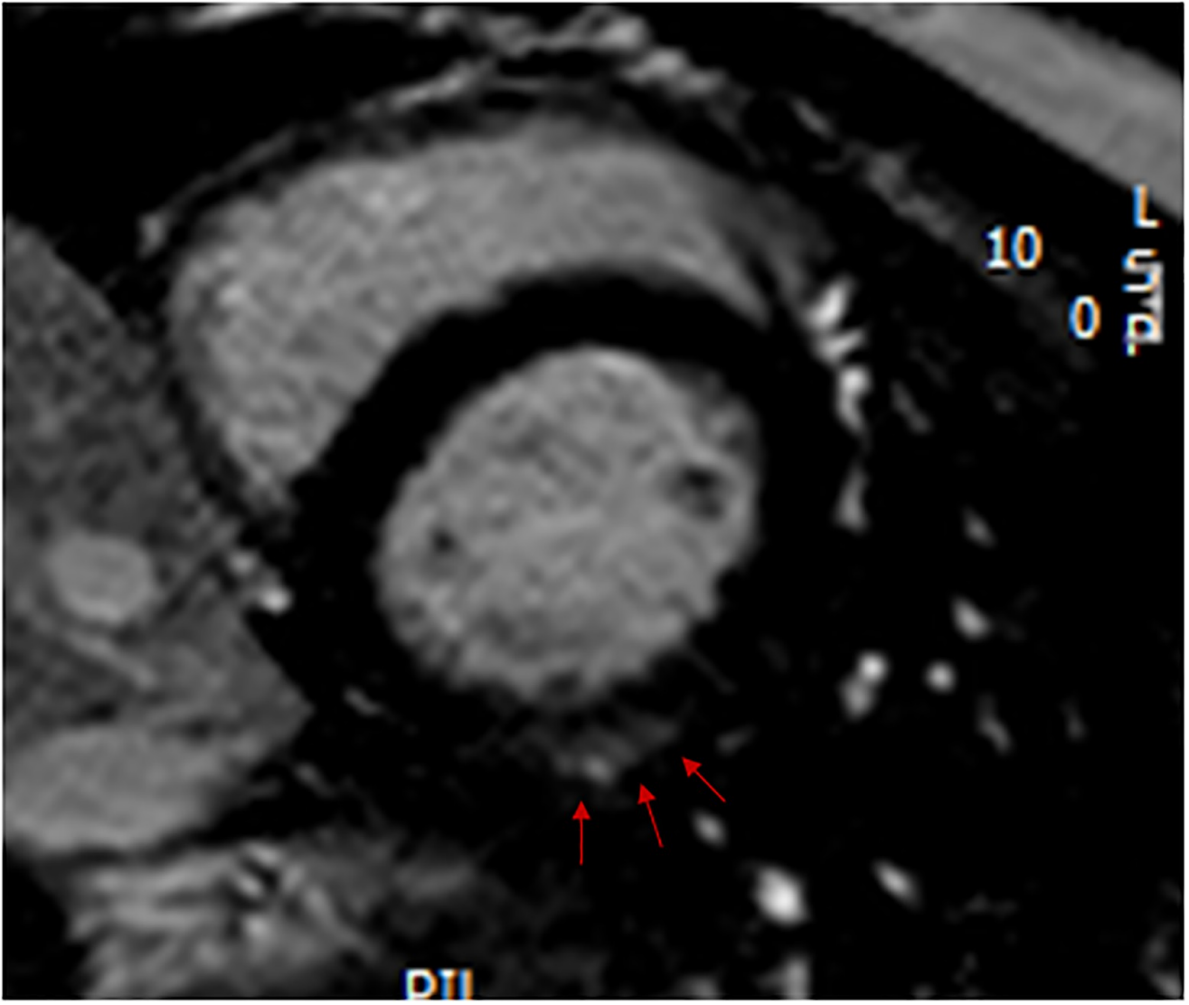
A cardiac magnetic resonance image showing a short-axis section of the left ventricle, with the red arrows showing mid myocardial late gadolinium enhancement in the basal segment of the inferolateral wall.

**Fig 2:**
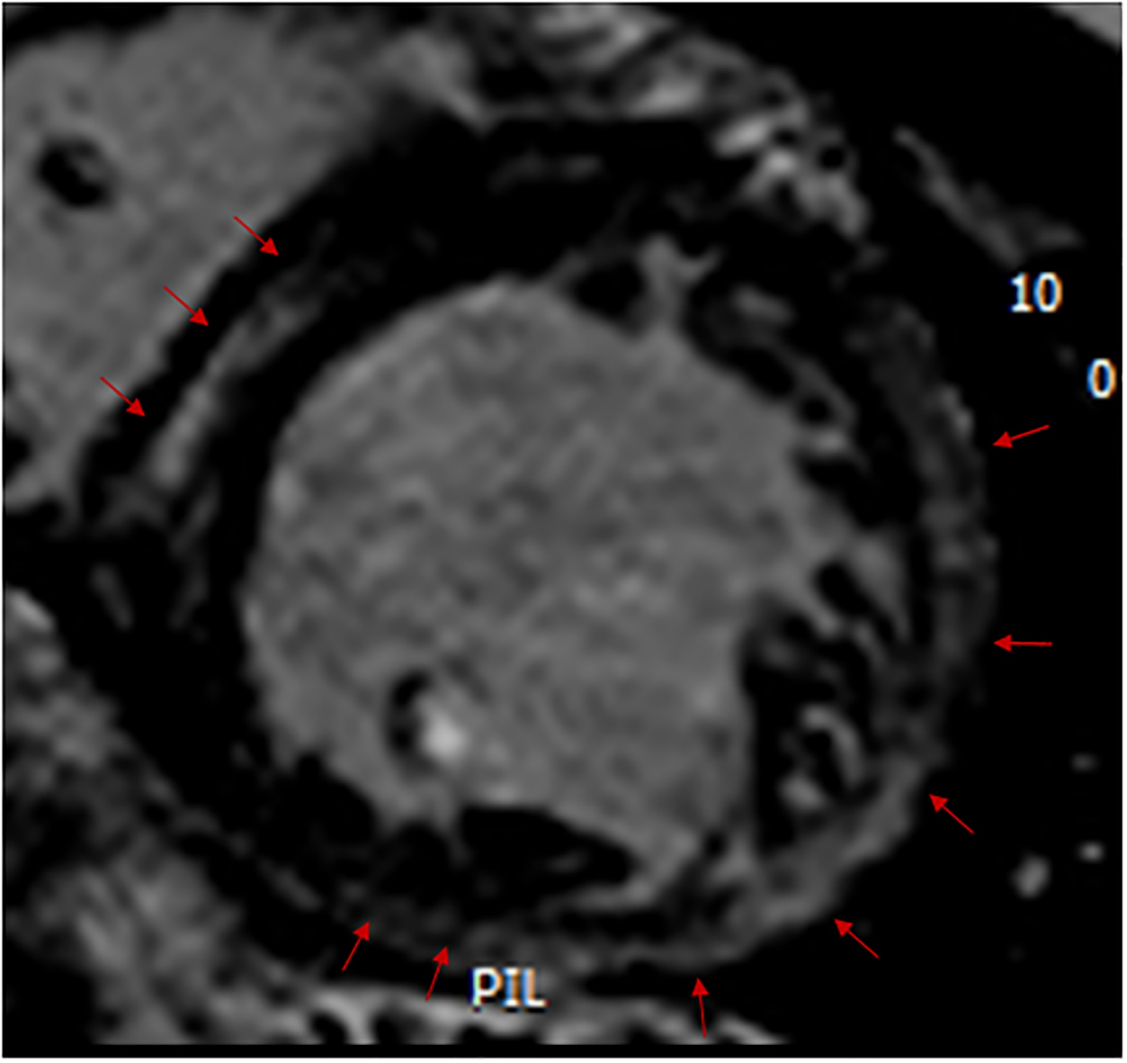
A cardiac magnetic resonance image showing a short-axis section of the left ventricle, with the red arrows on the left showing mid myocardial late gadolinium enhancement in the septal wall and the red arrows to the right of and underneath the ventricle showing epicardial late gadolinium enhancement in the anterolateral and inferolateral walls.

LGE was present in 19 of the 92 (21%) subjects who had a contrast injection. One of these 19 only had LGE at an insertion point. The number of segments involved in the other 18 subjects ranged from 2–14 (median of 3). Examples of LGE in two of these subjects are shown in Figs 1 and 2. The most common site for LGE was mid myocardium, but there were also segments with a subepicardial LGE location. There was only one subject in whom there was evidence of transmural LGE, this being a subject with a reduced LVEF and LGE in 14 segments. The walls most frequently involved in the 18 subjects were the anterolateral and inferolateral walls. Only two subjects had LGE in the septum. LGE was present in 20% of children and 21% of adults. In subjects with a LVEF ≥55%, there were 14/83 (17%) with LGE and in subjects with a LVEF<55% there were 5/9 (56%) with LGE. If more than 4 segments had LGE there was always a reduced LVEF. In logistic regression analysis of all subjects, a reduced LVEF was a predictor of LGE presence (p = 0.014), and there were no additional contributions to the prediction of LGE by sex, age group, GAA1, AOS, log SDur, age, LVEDVI or LVMI (p>0.10 for all). Similarly, in subjects with a normal LVEF, none of sex, age group, GAA1, AOS, log SDur, age, LVEDVI or LVMI were predictors of the presence of LGE (p>0.10 for all).

The 74 subjects without LGE were also investigated for an association of reduced LVEF with native T1 time and ECV. A reduced LVEF was not a predictor of native T1 time after adjustment for sex, heart rate and age group (p>0.50). However, a reduced LVEF remained a predictor of an increased ECV after adjustment for study site, age, sex and age group p = 0.04). In adult subjects with a preserved LVEF (n = 48) and without LGE, after adjustment for sex, GAA1 was still an independent predictor of native T1 time (β = 0.33, p = 0.014) and after adjustment for study site and log SDur, GAA1 was still an independent predictor of ECV (β = 0.35, p = 0.009).

## Discussion

Standard CMR measurements of LVM and LVEDV, in conjunction with T1 mapping techniques and LGE, were utilised in this study to quantify LV structure and ultrastructure in subjects with FRDA who were homozygous for GAA expansions in the *FXN* gene. The relationships of FRDA genetic severity (GAA1) with macroscopic LV structure (LVM and LVEDV) and LV ultrastructure (native T1 time, ECV and LGE) were also investigated. Positive correlations of GAA1 with native T1 relaxation time and ECV in FRDA subjects with a normal LVEF suggest an association between the degree of frataxin deficiency and the percentage of diffuse interstitial LV myocardial fibrous tissue. That these positive correlations were independent of changes in LVM and LVEDV suggests that T1 mapping techniques provide information about the LV myocardium which is at least partly independent of the recognised LV macroscopic structural changes of FRDA. The marker of myocardial replacement fibrosis, LGE, was found in only 21% of subjects with FRDA and there was substantial individual variation in the number of LV segments involved. LGE was more frequent in subjects with a reduced LVEF, but in subjects with a normal LVEF LGE was not associated with GAA1, LVEDVI or LVMI. The absence of predictors of LGE suggests that there is a different, and possibly idiosyncratic, mechanism underlying the development of replacement fibrosis compared to diffuse fibrosis in the early stages of cardiac disease in FRDA.

In multivariate models of LVEDV and LVM, BSA was the most important predictor of both variables, and male sex was an independent predictor of a larger LVM in adults. The contribution of male sex independently of BSA to a higher LVM is consistent with what is found in healthy subjects and also with previous CMR and echocardiographic findings in subjects with FRDA and a normal LVEF [11, 15]. The findings of the current study again highlight the importance of taking both body size and sex into account when categorising LV mass in FRDA, something which has not been performed in some previous studies [4–6]. The finding of an inverse relationship of LVM with age in the present cross-sectional study is consistent with a previous echocardiographic study [15] and two CMR studies in FRDA [11, 24]. This observation is at present unexplained and could represent a genuine reduction in LVM during disease progression [29], survivor bias, or a combination of both processes. In the present study, in the absence of age in the model of LVM, log SDur was not a predictor of LVM, this finding not supporting the hypothesis of a decrease in LVM during disease progression in FRDA.

In the present study LVM was increased above the normal sex, age-group and BSA adjusted ranges in 34% of children and 16% of adults. Meyer et al performed CMR in 41 adults with FRDA, 3 of whom had a LVEF <50%, and reported an increase in LVMI in 29% of subjects [11]. While this frequency seems substantially higher in comparison to the adults in the present study, the threshold for increased LVMI used by Meyer et al was lower, sex differences were not taken into consideration, and the LVM method used was different to the present study as it included papillary muscles in the LV volume rather than in the LVM. Rajagopalan et al also reported an increase in LVMI in FRDA compared to control subjects in a CMR study which included adults and children with a normal LVEF. However, the percentage of subjects with a LVMI above the normal range was not reported in this study, nor was there differentiation made between the LVMI in males and females [28]. An increase in LVMI based on echocardiography in subjects with a LVEF >50%, this study including some subjects from the current study at an earlier time point, also found higher percentages of age group and sex-adjusted LVMI compared to the present study (47% of children and 24% of adults) [15]. However, differences in the categorization of LVMI by CMR and echocardiographic studies is likely to reflect, at least in part, differences in both accuracy and reproducibility of the two techniques [39].

More common than an increase in LVM in the present study was the presence of an age-group, sex and BSA adjusted LVEDV less than the lower limit of the normal range, this being found in 39% of subjects (42% of adults and 30% of children). Thus, a low LVEDV was more common in adults than children, whereas a high LVM was more common in children than adults. Rajogapalan et al also reported a lower LVEDV using CMR in FRDA compared to control subjects, but did not adjust for age group, sex or BSA in their study [28]. Our finding is consistent with echocardiographic studies which have reported that the left ventricle is smaller in adults with FRDA and a normal LVEF when compared to age and sex-matched control subjects [13, 14]. The current study therefore provides additional evidence that the increases in wall thickness and LVM in FRDA are associated with a negative LV cavity remodelling process, as has also been reported in the sarcomeric hypertrophic cardiomyopathies [40]. With the combination of an increased LVMI and reduced LVEDVI in our cohort, an increase in LVMVR was inevitable, and in subjects with a LVEF ≥55%, the LVMVR was higher than the normal range in 89% of subjects in this study. Not predictable given the different patterns of increased LVMI and decreased LVEDVI in adults and children, was that the LVMVR in FRDA was higher in children than adults.

To the best of our knowledge this is the largest study of LGE in FRDA, it may be the only study to assess the incidence in LGE in a group including both adults and children, and it is also the only study to investigate the predictors of the presence of LGE in FRDA. LGE was only present in 21% of the subjects in the current study (age range 10–49 years, SDur range up to 36 years), and in contrast to the LVEDVI and LVMI differences between adults and children, this percentage was similar in adults and children. LGE was more common in subjects with a reduced LVEF, but it was not present in all subjects with a reduced LVEF. LGE location was mostly in the mid myocardium but there was also subepicardial involvement, whereas transmural LGE involvement was rare. The percentage of subjects with LGE in previous studies in FRDA has been variable, and both higher and lower percentages than those of the current study have been reported. Raman et al reported LGE in 58% of 26 adult subjects with a normal LVEF (age range 18–57 years, SDur not reported) [22]. Weidemann et al reported LGE in 66% of 32 subjects (age 33±13 years, number of children and SDur not reported), 25% of whom had a LVEF <55% [6]. All those with a reduced LVEF were positive for LGE, whereas 13/25 (52%) with a normal LVEF had LGE. That children with FRDA can develop LGE was also previously reported in 3 children with a normal LVEF by Mavrogeni et al [23]. Takazaki et al reported only 15% LGE positivity in 27 subjects with a normal LVEF (age 28±10 years, number of children not reported, SDur = 13±8 years) [24]. We can provide no explanation for the substantial variation in LGE positivity in the different studies, particularly given the absence of important details about the age groups of the subjects and the SDur for some of the cohorts. On the other hand, our finding that the mid myocardium was the most common site for LGE is consistent with the findings of both Raman et al [22] and Weidemann et al [6].

GAA1 is known to be inversely associated with frataxin levels [25, 26] and with the AOS [15], and to be positively associated with the severity of FRDA cardiac involvement [4, 41]. In the present study, a larger GAA1 was associated with a larger LVM in adults independently of both sex and body size. Positive correlations of GAA1 with LVMI have been reported in some [9], but not other [12, 15], echocardiographic studies, and have not been reported in previous CMR studies [11, 28]. The discrepancies between studies regarding the relationship of GAA1 with LVM could be because the correlation of GAA1 with LVM is relatively weak, that the studies have been small, and/or that LVMI is less accurately calculated with echocardiography compared to CMR [42]. Although LVEDV was smaller than the normal range in 39% of subjects in the current study, GAA1 was not a predictor of a lower LVEDV. A weak negative correlation of GAA1 with LVEDV has been reported previously in one echocardiographic study [15].

We also examined the relationship of AOS with LV variables in this study, although issues related to collinearity needed to be considered given the expected and observed inverse correlation of GAA1 with AOS (r = -0.60). In adults, LVM was positively correlated with GAA1 and negatively correlated with AOS, but these associations were not independent. In contrast, in adults, LVEDV was positively correlated with AOS despite not being correlated with GAA1, suggesting that the small contribution of AOS to the prediction of LVEDV was independent of GAA1. The role of SDur was also investigated in this study, but a longer SDur was only found to be an independent predictor of a smaller LVEDV in children and a larger ECV in adults.

After adjustment for study site, the ECV was higher in subjects with a reduced LVEF compared to those with a normal LVEF, and in the group of subjects with a normal LVEF, it was higher in children than adults. A recent study reported on ECV in FRDA subjects using a 3.0T machine [25], finding it to be 0.36±0.05 in 27 subjects with FRDA and a normal LVEF who were of age 28±10 years, this being substantially higher than the value of 0.25±0.02 in the 10 control subjects in that study. In contrast, in the current study using a 1.5T machine the ECV in subjects with a normal LVEF was only 0.271±0.045 at the Philadelphia site and 0.242±0.030 at the Melbourne site. One possible explanation for the much higher average ECV in the Takazaki et al study compared to the present study is that it has been reported that the native T1 time is longer when assessed by a 3T compared to a 1.5T machine [38]. Moreover, the type of acquisition sequence has been shown to affect both native T1 time and ECV [37]. In addition, the comparison with control subjects in the study of Takazaki et al was confounded by the small size of the control group (n = 10), with the imaging performed at a separate site and on a different machine, and the adequacy of age and sex matching uncertain.

The independent positive associations of GAA1 with both native T1 time and ECV in the present study provide evidence supporting a causal relationship between the severity of frataxin deficiency and an increased ratio of interstitial fibrous/cardiomyocyte volume in FRDA. However, there were no control subjects in the present study and there is only limited data in the literature about normal sex and age values for native T1 and post contrast T1 times and ECV based on the methodology used in the present study. Our study can therefore provide no definitive evidence that native T1 time and ECV are increased in FRDA, or how common abnormalities in native T1 time and ECV might be in FRDA. That sex and age based normal values are essential for this purpose is supported by studies in healthy subjects in which males have a lower native T1 time and ECV than females [33–38] and where there is at least a trend, possibly modified by sex, to a lower native T1 time with older age [33–36]. In the present study the native T1 time was lower in males, and the ECV was lower in adult males with a normal LVEF, suggesting that similar to healthy subjects, in FRDA there are not only sex effects on LVMI but also sex effects on the myocardium at the ultrastructural level.

There is the potential for clinical and ongoing research roles for T1 mapping in FRDA given that both the native T1 time and ECV can provide information about the LV myocardium prior to a reduction of LVEF, and that this information is at least partly independent of the FRDA-associated LV structural changes. However, the nature, timing and speed of the progression of changes in T1 mapping variables in FRDA are unknown, and reproducibility and robustness of T1 mapping techniques for serial testing are uncertain. In the present study there were differences in post contrast T1 time and ECV based on the study site even though the machines, protocols and analysis software were similar at the two sites, and the explanation for this difference remains uncertain. On the other hand, native T1 time did not appear to be influenced by the study site, and this simpler technique provides the additional advantages for the subject in that it does not require gadolinium injection and there is also less scanning time required.

There are a number of limitations of this study. The number of children in the study was fewer than the number of adults and this resulted in lower statistical power to detect effects of FRDA on CMR variables in children. All studies in FRDA are at least partly confounded by recruitment being limited to subjects who have presented with symptoms and also by the absence of those subjects who have had disease progression and died as a result of the disease. For a CMR study in FRDA there is the additional limitation that some subjects were ineligible because of the presence of spinal rods inserted for the treatment of FRDA-associated scoliosis. An appropriate control group for T1 mapping would have enabled determination of the frequency of T1 mapping variable abnormalities in FRDA. However, this was not feasible as it would have required substantial numbers at both sites to adequately match the age range and sex mix of the FRDA subjects. Moreover, it would be difficult to justify the use of intravenous gadolinium injection in normal children, or indeed to even be able to recruit healthy children for such a study. A further limitation was that the methods for GAA estimation differed and there is therefore a possibility of systematic differences in GAA repeat lengths between the different assays. However, this possibility is more likely to lead to false negative than false positive findings. Moreover, adjustment for potential differences in techniques between the 2 institutions has been addressed in part in the regression analyses in which GAA repeat length was tested an independent variable by the inclusion of the institution as a dummy variable. Although there were differences in the prediction of ECV and post GAD T1 time based on institution there were no differences based on institution in the prediction of native T1 time. This suggests that the institutional differences could have been related to the post GAD data but does not support an independent contribution from differences in GAA estimation. Lastly, there is uncertainty regarding the mechanistic significance of some of the regression analysis findings due to collinearity between GAA1 and AOS and also between age and SDur. That there were independent effects of age from SDur and vice versa was suggested by the evidence that age, but not SDur, was an independent predictor for LVM in adult subjects with a normal LVEF and that SDur, but not age, was an independent predictor of ECV in adult subjects with a normal LVEF.

In conclusion, there is an association between diffuse interstitial LV myocardial fibrosis and genetic severity in FRDA, with this effect being independent of the FRDA-associated LV changes in LVEDVI and LVMI. Localised replacement fibrosis was found in a minority of subjects with FRDA, this minority including both children and adults, and subjects with and without a reduction of LVEF. In contrast to T1 mapping variables, LGE in subjects with a normal LVEF was not associated with genetic severity, and was also not associated with LVM or LVEDV, consistent with the development of LGE in FRDA having an idiosyncratic element. There is the potential for roles of both T1 mapping and LGE in the assessment and monitoring of the cardiomyopathy of FRDA, but information will be required about their prognostic significance, and more data will also be required regarding the reproducibility of T1 mapping variables.

## Supporting information

S1 File Anthropomorphic and cardiac magnetic resonance data in subjects with Friedreich ataxia. (XLSX)

## Abbreviations

AOS: age at onset of symptoms
BSA: body surface area
CMR: cardiac magnetic resonance
ECV: left ventricular myocardial extracellular volume fraction
FRDA: Friedreich ataxia
GAA1: number of GAA repeats in the smaller allele of the *FXN* gene
LGE: late gadolinium enhancement
LV: left ventricular
LVEDV: left ventricular end-diastolic volume
LVEDVI: left ventricular end-diastolic volume indexed to body surface area
LVEF: left ventricular ejection fraction
LVESV: left ventricular end-systolic volume
LVM: left ventricular mass
LVMI: left ventricular mass indexed to body surface area
SDur: symptom duration
SV: stroke volume
